# Supplementary figures and images for: Long-term associative memory in rats: Effects of familiarization period in object-place-context recognition test
Source: PLoS One. 2021 Jul 30;16(7):e0254570. doi: 10.1371/journal.pone.0254570 (PMC8323955; doi:10.1371/journal.pone.0254570)

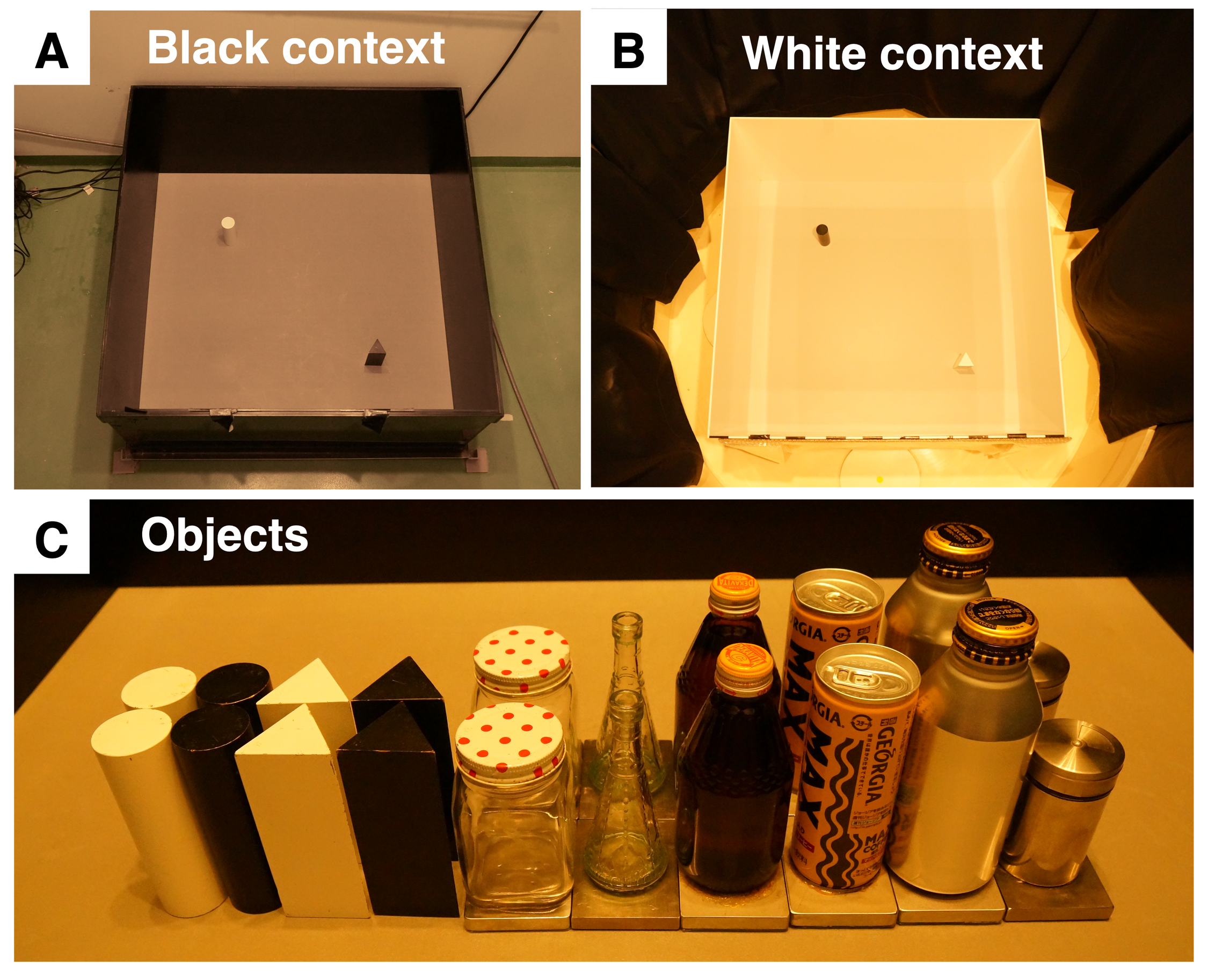

Supplement: S1 Fig — Apparatus of black context (A), white context (B), and objects (C) used in the experiments. (TIF) [file pone.0254570.s001.tif]

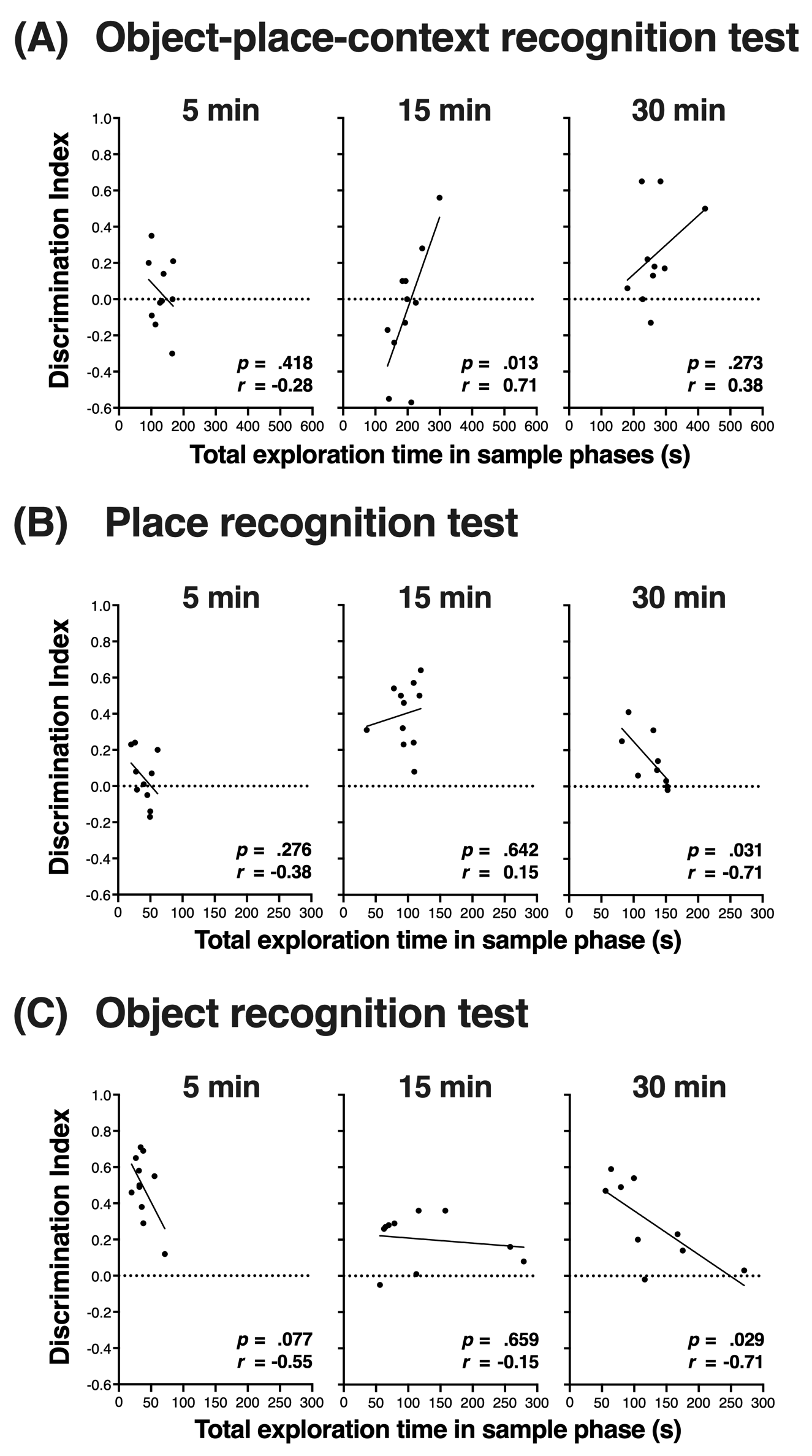

Supplement: S2 Fig — Correlations between exploration time in sample phase and discrimination index in test phase for the object-place-context (A), place (B), object (C) recognition tests. (TIF) [file pone.0254570.s002.tif]
